# β-adrenergic receptor-induced E-S potentiation in the dorsal and ventral hippocampus

**DOI:** 10.3389/fnsyn.2024.1511485

**Published:** 2024-12-20

**Authors:** George Trompoukis, Athina Miliou, Costas Papatheodoropoulos

**Affiliations:** Laboratory of Physiology, Department of Medicine, University of Patras, Patras, Greece

**Keywords:** hippocampus, dorsoventral, norepinephrine, β adrenergic receptors, E-S potentiation, long-term plasticity, intrinsic excitability, LTP

## Abstract

β-adrenergic receptors (β-ARs) play a critical role in modulating learning, memory, emotionality, and long-term synaptic plasticity. Recent studies indicate that β-ARs are necessary for long-term potentiation (LTP) induction in the ventral hippocampus under moderate synaptic activation conditions that do not typically induce LTP. To explore potential dorsoventral differences in β-AR-mediated effects, we applied the β-AR agonist isoproterenol (10 μM, 30 min) to dorsal and ventral hippocampal slices, recording field excitatory postsynaptic potentials (fEPSPs) and population spikes (PSs) from the CA1 region. Isoproterenol induced robust, long-lasting PS increases, with effects three times greater in the dorsal compared to the ventral hippocampus. Isoproterenol did not significantly affect fEPSP in either segment of the hippocampus, leading to strong excitatory-to-spike (E-S) potentiation—twice as large as that in the ventral hippocampus. E-S potentiation was not associated with significant paired-pulse inhibition changes in either hippocampal segment. These differences do not appear to result from β1-AR expression levels, as they are comparable across dorsal and ventral hippocampal regions. Overall, the findings suggest that β-AR activation enhances the dorsal hippocampus’s role during stress, facilitating heightened alertness, rapid spatial information processing, and effective navigation necessary for “fight-or-flight” responses.

## 1 Introduction

The noradrenergic system is fundamentally involved in many brain functions including arousal, attention, emotional regulation, responses to stress, cognitive flexibility, and learning and memory (Aston-Jones and Cohen, 2005a; Sara, 2015). Furthermore, norepinephrine, acting on β adrenergic receptors (β-ARs), plays a critical role in the processing of emotionally charged memories and profoundly modulates activity-dependent long-term synaptic plasticity (O'Dell et al., 2015; Hagena et al., 2016). Synaptic plasticity, i.e., activity-dependent persistent changes in synaptic efficacy, represents a fundamental mechanism for information storage in the nervous system and is considered a key neurobiological substrate for learning and memory that help brain circuits to adapt to new behavioral demands (Eichenbaum, 1996; Takeuchi et al., 2014; Sweatt, 2016; Humeau and Choquet, 2019).

Activation of β-ARs in the hippocampus facilitates long-term synaptic potentiation (LTP) induced by high-frequency stimulation or patterned stimulation at the theta frequency (Thomas et al., 1996; Katsuki et al., 1997; Brown et al., 2000; Straube et al., 2003; Gelinas et al., 2008; Jedrzejewska-Szmek et al., 2017; Jami et al., 2023), and is deeply involved in modulating hippocampus-dependent memory (Ji et al., 2003; Hansen and Manahan-Vaughan, 2015; O'Dell et al., 2015; Gao et al., 2016; Hagena et al., 2016; Mello-Carpes et al., 2016). Emerging evidence suggests that β-AR activation may have region-specific effects on LTP within the hippocampus, raising questions about the differential contribution of these receptors to synaptic plasticity across its dorsal and ventral domains. It has recently been shown that activation of β-ARs, either by isoproterenol or endogenous norepinephrine, under conditions of patterned theta-frequency stimulation, facilitates the induction and stabilization of LTP more in the ventral than the dorsal CA1 region of the hippocampus. This suggests that β-ARs in the ventral hippocampus may play a critical role in the detection and storage of emotionally salient information, such as novelty detection, while β-ARs in the dorsal hippocampus may contribute to the consolidation of information under conditions of particularly strong inputs (Papaleonidopoulos and Papatheodoropoulos, 2018; Jami et al., 2023).

Typically, changes in synaptic inputs received by a neuron are accompanied by proportional changes in the output of a neuron, i.e., the action potentials produced by the neuron in response to the synaptic input and ultimately convey information through the nervous system. However, long-lasting changes in synaptic inputs can be accompanied by disproportionately large changes in neuronal output, suggesting that long-lasting changes in excitability may represent an important mechanism for information storage in the nervous system (Zhang and Linden, 2003; Debanne et al., 2019). The phenomenon of persistently increased ability of a neuron to fire action potentials in response to a given synaptic input was described already in the first reports of LTP in hippocampal dentate gyrus (Bliss and Lomo, 1973), was termed EPSP-spike (E-S) potentiation (Andersen et al., 1980) and apparently reflects an increased electrical coupling between the synaptic inputs in the dendrites and the somatic region. Previous evidence has shown that E-S potentiation may engage several mechanisms, including an increase in intrinsic excitability (Hess and Gustafsson, 1990; Asztely and Gustafsson, 1994; Jester et al., 1995; Fink and O'Dell, 2009), or a long-term reduction in inhibitory synaptic transmission (Abraham et al., 1987; Chavez-Noriega et al., 1989; Lu et al., 2000; Chevaleyre and Castillo, 2003). Alternatively, it has been postulated that a long-term alteration in the balance between excitation and inhibition (E/I) may also lead to E-S potentiation (Abraham et al., 1987; Chavez-Noriega et al., 1989; Marder and Buonomano, 2004). E-S potentiation has been typically studied using high-frequency stimulation or theta frequency stimulation of the afferent inputs (Bliss and Lomo, 1973; Andersen et al., 1980; Wilson et al., 1981; Fink and O'Dell, 2009; Wójtowicz and Mozrzymas, 2014). However, E-S potentiation can be also induced by neuromodulators (Chevaleyre and Castillo, 2003; Mlinar et al., 2008). Results from early studies in the dentate gyrus of the hippocampus have suggested that norepinephrine can induce potentiation of neuronal excitability in the absence of potentiation in the excitatory synaptic transmission (Neuman and Harley, 1983; Lacaille and Harley, 1985; Heginbotham and Dunwiddie, 1991), suggesting that multiple mechanisms and signaling pathways may be recruited in response to different patterns of neuronal activity to contribute to E-S potentiation.

A basic role of norepinephrine in the brain is to dynamically modulate the balance between excitation and inhibition in a neuronal network leading to a change in the input–output relationship between synaptic inputs and neuronal output (Aston-Jones and Cohen, 2005b; Mather et al., 2016). Activation of β-ARs increase the E/I balance by enhancing the excitability through downregulation of potassium channels (Hoffman and Johnston, 1999; Faber et al., 2008; Liu et al., 2017). The β-AR-induced increase in excitability may also be a crucial mechanism underlying the increased efficacy of β-ARs in LTP induction by theta-burst stimulation in the ventral hippocampus (Papaleonidopoulos and Papatheodoropoulos, 2018; Jami et al., 2023). However, the role of β-ARs in E-S potentiation in the CA1 hippocampal region has never been studied before comparatively in the dorsal and ventral hippocampus.

In this study, we aimed to investigate whether activation of β-ARs alone can recapitulate the dorsoventral differences in long-term synaptic plasticity observed in response to combined moderate adrenergic activation with increased presynaptic activity, and whether the induction of synaptic plasticity is accompanied by long-lasting changes in neuronal network excitability. By elucidating these regional differences, this research could provide valuable insights into how adrenergic signaling contributes to the functional specialization of hippocampal subregions and their roles in cognition and emotion.

## 2 Materials and methods

### 2.1 Slice preparation

In this study we used slices from the dorsal and the ventral hippocampus of adult (3–4 months) male Wistar rats. Rats were obtained from the Laboratory of Experimental Animals of the Department of Medicine, University of Patras (license no.: EL-13-BIOexp-04). The animal treatment and all experimental procedures used in this study were performed in compliance with the European Communities Council Directive Guidelines for the care and use of Laboratory animals (2010/63/EU – European Commission), and the experimental protocol has been approved by the Protocol Evaluation Committee of the Department of Medicine of the University of Patras and the Directorate of Veterinary Services of the Achaia Prefecture of Western Greece Region (reg. Number: 187531/626, 26/06/2018). Rats were maintained under standard conditions of temperature (20–22°C) and light–dark cycle (12/12 h), and they were free access to food and water. Hippocampal slices were prepared as previously described (Trompoukis and Papatheodoropoulos, 2020). Briefly, after scarifying rats by decapitation under deep anesthesia with diethyl-ether, the brain was removed, placed in chilled artificial cerebrospinal fluid (ACSF) at 2–4°C, equilibrated with 95% O_2_ and 5% CO_2_ gas mixture. The composition of ACSF was (in mM): 124 NaCl, 4 KCl, 2 CaCl_2,_ 2 MgSO_4,_ 26 NaHCO_3_, 1.25 NaH_2_PO_4_ and 10 glucose and a pH = 7.4. After removing the two hippocampi, slices 500 μm thick were prepared from the dorsal and ventral end of the hippocampus - extending between 0.5 mm and 4.0 mm from each end- by cutting the hippocampus transversely to its long axis using a McIlwain tissue chopper. Slices were transferred to an interface type recording chamber where they were maintained for the rest of the experiment continuously perfused with ACSF at a rate of ~1.5 mL/min and humidified with 95% O_2_ and 5% CO_2_ gas mixture at a temperature of 30.0 ± 0.5°C. Slices were allowed to recover for about 1.5 h before start stimulation and recording.

### 2.2 Electrophysiology and data analysis

We recorded evoked field potentials from the middle CA1 region of the hippocampus following stimulation of Schaffer collaterals. Specifically, we made simultaneous recordings of field excitatory postsynaptic potentials (fEPSPs) and population spikes (PSs) from the stratum radiatum and the stratum pyramidale, respectively, using 7 μm-thick carbon fiber electrodes (Kation Scientific, Minneapolis, USA). In the experiments of 60-min application of ISO (described in section 6.3), recordings from the stratum pyramidale were used to measure both PS and fEPSP. Stimulation consisted of constant current pulses of 100 μs in duration and variable amplitude delivered using a home-made bipolar platinum/iridium wire electrode with a wire diameter of 25 μm and an inter-wire distance of 100 μm (wire was purchased from World Precision Instruments, USA). The stimulation and recording electrodes were 200–400 μm apart. The rate of baseline stimulation was 1/30 s. Field potentials were recorded using Neurolog systems (Digitimer Limited, UK), digitized using a CED 1401-plus interface and the Signal5.09 software (Cambridge Electronic Design, Cambridge, UK). Singal was amplified X 500, band-pass filtered at 0.5 Hz–2 kHz, digitized at 10 kHz, and stored on a computer disk for off-line analysis. fEPSP was quantified by the maximum slope of its rising phase. Furthermore, PS was quantified by its amplitude measured as the length of the projection of the minimum peak on the line connecting the two maximum peaks of the PS waveform. We quantified the paired-pulse ratio (PPR) of synaptic responses evoked by paired-pulse stimulation (fEPSP2/fEPSP1), as well as the paired-pulse inhibition (PPI) by measuring the ratio between the PS evoked by the second pulse and the PS evoked by the first pulse of a pair (PS2/PS1 ratio). Paired-pulse stimulation was applied at an inter-pulse interval of 20 ms. We used the specific agonist of β-ARs (+)-isoproterenol (+)-bitartrate salt (ISO, 10 μΜ).

### 2.3 Immunoblotting

The CA1 region from the dorsal and the ventral hippocampal slices were stored at −80 °C for protein expression analysis. Tissue was homogenized with sonication in 200 μL 1% SDS with 1% protease inhibitors (Sigma Aldrich) for 2 × 15 s. Protein concentration was determined for each sample using the NanoDrop™ 2000/2000c Spectrophotometer (Thermo Scientific). Protein homogenates (30 μg of protein per lane) were subjected to SDS-PAGE electrophoresis on 10% polyacrylamide gels for 30 min at 80 V and 1 h at 120 V. Proteins were transferred to PVDF membrane (Amersham Hybond) at 400 mA for 90 min, and the membranes were incubated for 1 h at room temperature in PBS containing 0.1% Tween-20 (PBST) and 5% nonfat dried milk to block non-specific sites. The membranes were next incubated overnight at 4 °C with the following primary antibodies diluted in 3% PBST: rabbit anti-ADRB1 polyclonal antibody (1:500 #DF3511, Affinity Biosciences) and mouse anti-beta-actin monoclonal antibody (1:10000 #MA5-11869, Thermo Fisher Scientific). The blots were rinsed with PBST and then incubated with secondary HRP-conjugated goat anti-rabbit IgG antibody (1:20000 #7074, Cell Signaling Technology) and with anti-mouse (1:15000 #A16084, Thermo Fisher Scientific). The bands were visualized on ChemiDoc MP P (BioRad, California, USA) by enhanced chemiluminescence (Immobilon ECL Ultra Western HRP Substrate, # WBULS0500, Millipore Sigma, Burlington, MA, USA) for 1–10 min. Optical density measurements from each band were defined as ROD units with the ImageLab 6.1. The ROD of each band was quantified relative to the ROD of beta-actin, which served as the gel-loading control. Then, the ratio (ROD of protein of interest / ROD of beta-actin) was normalized with the same ratio of an internal sample, which was loaded in all the gels.

### 2.4 Statistics

We used the Shapiro–Wilk test to assess the normality of the variable distributions. For variables that were normally distributed, drug effects within each hippocampal segment were statistically evaluated using paired *t*-tests, while comparisons of drug effects between the two hippocampal segments were analyzed using independent *t*-tests, since in all these cases we found normally distributed variables. The Levene’s test was used to assess the equality of population variances. To examine drug effects in each hippocampal segment, we also used the repeated measures General Linear Model (GLM) or the corresponding non-parametric test (Friedman test). Baseline stability during the control period was assessed via linear regression analysis of the pre-treatment data. Slices that showed significant trend during the baseline were occluded from further analysis. Details regarding the number of slices and rats used in each experimental condition are provided.

## 3 Results

### 3.1 Effects of β-AR activation on fEPSP

**Figure 1:**
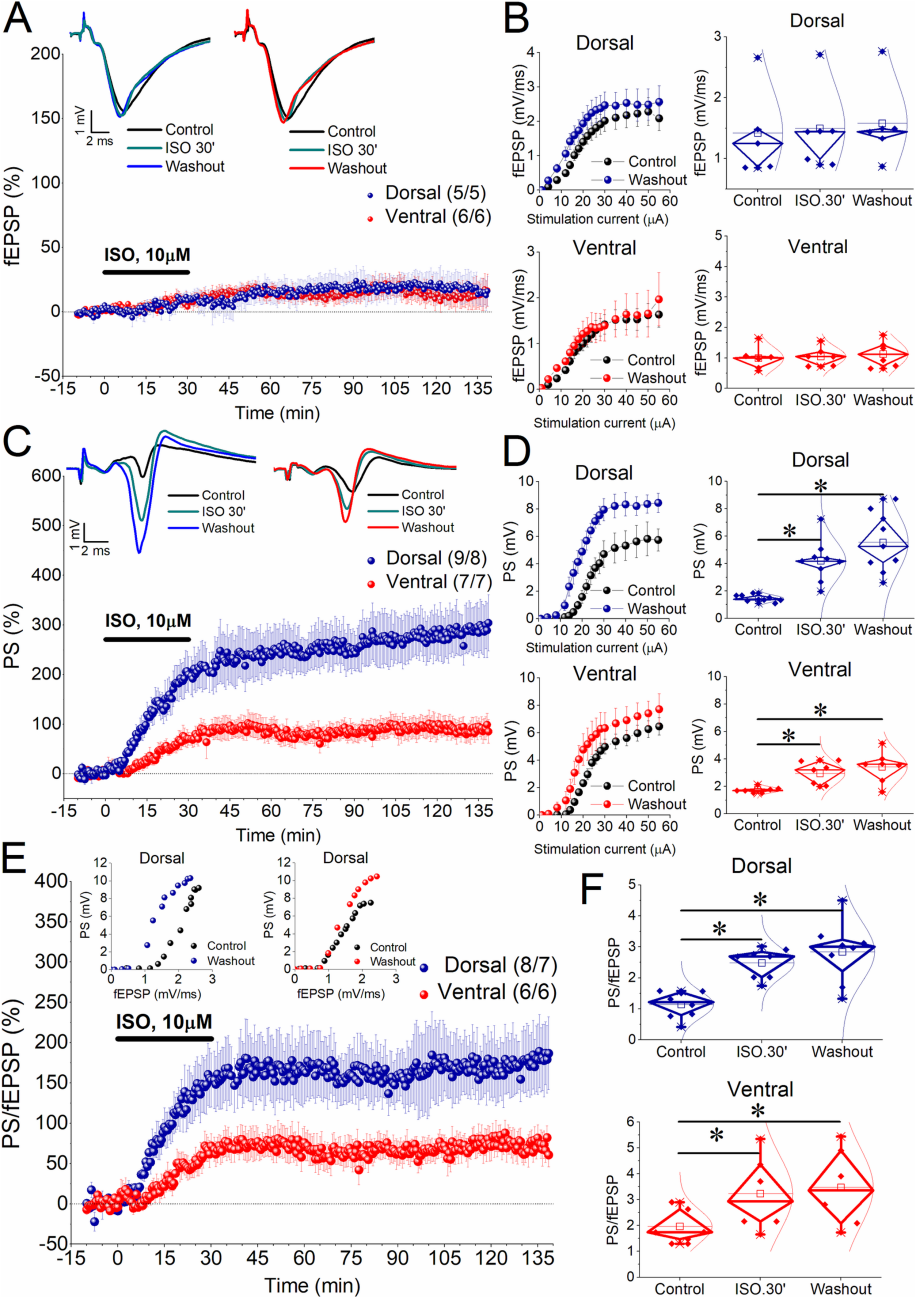
Induction of long-term enhancement of PS by 10 μM isoproterenol (ISO) applied for 30 min in dorsal and ventral hippocampal slices. **(A)** Time course of percent change of fEPSP showing that application of 10 μM ISO for 30 min does not significantly affect synaptic transmission in either segment of the hippocampus. Representative traces obtained during Control conditions, 30 min of ISO application and after washing out ISO are shown on the top of the graph. **(B)** Cumulative input–output curves between stimulation current intensity and fEPSP (left panel) under control conditions and after washing out ISO are shown on the left, while box plots of fEPSP obtained before perfusion with ISO (Control), at the end of ISO perfusion (ISO.30′), and after washing out ISO (Washout) are shown on the right. Data for the dorsal and ventral hippocampus are shown in the upper and bottom graphs, respectively. **(C)** Time course of percent change of PS showing that application of 10 μM ISO for 30 min induces long-term potentiation of neuronal excitation in the CA1 region of the dorsal and ventral hippocampus. Representative traces obtained during Control conditions, 30 min of ISO application and after washing out ISO are shown on the top of the graph. **(D)** Aggregate input–output curves between stimulation current intensity and PS obtained from the dorsal and ventral hippocampus (upper and bottom graphs, respectively), and corresponding box plots are shown. **(E)** Time course of percent change of PS/fEPSP ratio showing that application of 10 μM ISO for 30 min induces persistent potentiation of neuronal excitability (E-S potentiation) more in dorsal and ventral hippocampus. Examples of input–output relationship between fEPSP and PS are shown on the top. Note the characteristic leftward shift of this curve obtained from the dorsal hippocampus after washing out ISO compared with that under control conditions. **(F)** Box plots of the PS/fEPSP ratio obtained before perfusion with ISO (Control), at the end of ISO perfusion (ISO.30′), and after washing out ISO (Washout) are shown for the dorsal and ventral hippocampus (upper and bottom graphs, respectively). Numbers into parenthesis indicate the number of slices/rats used. Asterisks denote statistically significant differences at *p < 0.05, paired *t*-test and pairwise comparisons. Statistically not significant differences are not indicated. More detailed statistical results are described in the main text. Box plots in this and the next figure show the median with the 25th and 75th quartiles (diamond box), the mean, the 5th and 95th percentile (thick line through small box and whiskers, respectively), and individual data points including outliers.

We perfused slices from the dorsal and the ventral hippocampus with 10 μM isoproterenol (ISO) for 30 min while simultaneously recording fEPSP and PS from the CA1 region. We selected an increased concentration of ISO to simulate conditions of heightened adrenergic activation without intense glutamatergic input which is typically mimicked using high-frequency stimulation. This approach allowed us to compare the effects of high adrenergic activation alone with those seen under lower adrenergic activation combined with increased glutamatergic input, as examined in previous studies (Papaleonidopoulos and Papatheodoropoulos, 2018; Jami et al., 2023). We found that application of ISO for 30 min did not significantly affect fEPSP either in the dorsal (7.02 ± 3.9%, t_4_ = −1.8, *p* = 0.147) or the ventral hippocampus (8.85 ± 4.45%, t_5_ = −1.55, *p* = 0.183; Figures 1A,B). Furthermore, fEPSP did not show any significant change compared to baseline after washing out ISO, either in the dorsal [16.05 ± 13.39%, t_4_ = −1.42, *p* = 0.23; *F*_(2,10)_ = 1.408, *p* = 0.289] or the ventral hippocampus [16.04 ± 7.81%, t_5_ = −1.83, *p* = 0.127; *F*_(2,10)_ = 1.75, *p* = 0.223]. The percent changes of fEPSP were similar between the two hippocampal segments at 30 min ISO (t_9_ = −0.263, *p* = 0.8) and drug washout (t_9_ = 0.118, *p* = 0.91). The lack of a significant ISO effect on dorsal hippocampal fEPSP observed here appears to contrast with the previously reported enhancing action of ISO (Miliou et al., 2021). This discrepancy may be explained by the fact that relatively high concentrations of ISO, such as those used in the present study, may also activate α1 adrenergic receptors (Copik et al., 2015), which have been shown to inhibit excitatory synaptic transmission (Lippiello et al., 2015).

### 3.2 Effects of β-AR activation on PS

In contrast to the absence of a significant effect on fEPSP, ISO enhanced neuronal excitation both acutely and in the long-term in both the dorsal and ventral hippocampus. Specifically, application of ISO for 30 min significantly enhanced the PS in the dorsal (190.94 ± 36.96%, t_8_ = −5.58, *p* < 0.001) and the ventral hippocampus (66.89 ± 15.75%, t_6_ = −4.22, *p* = 0.006). The ISO-induced acute increase in the PS was significantly greater in the dorsal hippocampus compared with the ventral hippocampus (t_14_ = 2.79, *p* = 0.014); these results are consistent with previous findings obtained using 1 μM ISO (Miliou et al., 2021). Interestingly, the enhancement of PS persisted for at least 1 h and a half after washing out the drug, in both the dorsal hippocampus [278.26 ± 45.14%, t_8_ = −5.96, *p* < 0.001; *F*_(2,16)_ = 28.29, *p* < 0.001] and the ventral hippocampus [93.52 ± 21.69%, t_6_ = −4.28, *p* = 0.005; *F*_(2,12)_ = 13.94, *p* < 0.001] (Figures 1C,D). Accordingly, the long-term enhancement of the PS was three times stronger in the dorsal than in the ventral hippocampus (t_14_ = 3.36, *p* = 0.005).

### 3.3 β-AR-induced E-S potentiation

Next, we aimed to determine whether the enhancement of the PS reflects an increase in network excitability or if it is better explained by long-term enhancement in synaptic transmission, particularly in the dorsal hippocampus. Accordingly, we measured the PS/fEPSP ratio, which provides a reliable estimate of local network excitability.

We found that ISO significantly increased the PS/fEPSP ratio in both the dorsal and ventral hippocampus (Figures 1E,F). Specifically, a 30-min application of ISO enhanced the PS/fEPSP ratio by 148.04 ± 34.33% in the dorsal hippocampus (t_7_ = −8.28, *p* < 0.001) and by 61.02 ± 15.42% in the ventral hippocampus (t_5_ = −3.4, *p* = 0.019), (dorsal vs. ventral hippocampus, t_12_ = 2.06, *p* = 0.06). As with the PS, the PS/fEPSP ratio remained increased after washing out ISO, in both the dorsal hippocampus [169.17 ± 36.64%, t_7_ = −5.64, *p* < 0.001; *F*_(2,14)_ = 27.09, *p* < 0.001] and the ventral hippocampus [73.04 ± 14.66%, t_5_ = −3.98, *p* = 0.011; *F*_(2,10)_ = 13.32, *p* < 0.002]. Notably, the long-term increase in the PS/fEPSP ratio in the dorsal hippocampus was significantly greater - more than double - compared to the ventral hippocampus (t_9,2_ = 2.5, *p* = 0.034) (Figures 1E,F). Since ISO did not significantly affect fEPSP in either hippocampal segment, these findings indicate that activation of β-ARs alone - without requiring high-frequency stimulation of the Schaffer collaterals - induces E-S potentiation in the CA1 region of both the dorsal and ventral hippocampus, with a significantly greater magnitude in the dorsal hippocampus.

### 3.4 Effects of β-AR activation on PPR (fEPSP2/fEPSP1)

**Figure 2:**
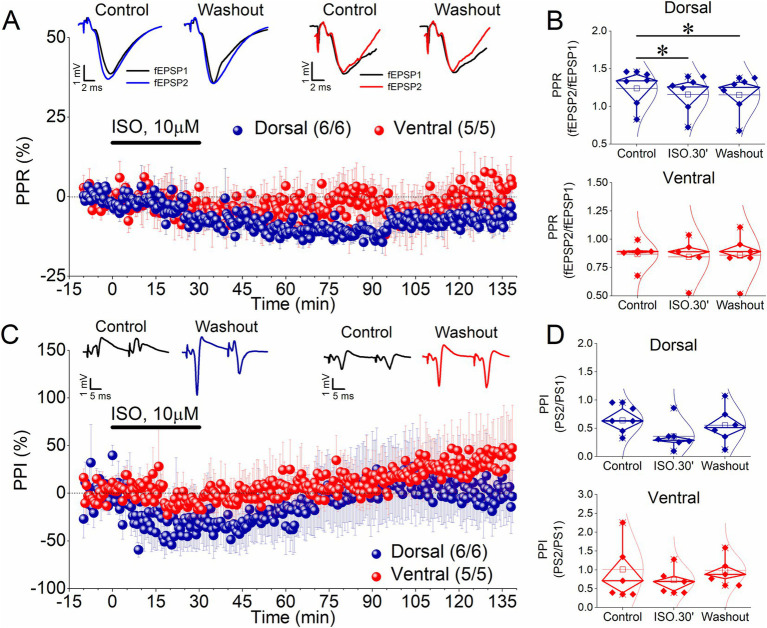
ISO induces a long-term reduction in the PPR (fEPSP2/fEPSP1 ratio) in the dorsal but not the ventral hippocampus, without affecting the PPI (PS2/PS1 ratio) in any segment of the hippocampus. **(A)** Time course of the percent change of the PPR in the dorsal and ventral hippocampus. Representative traces of paired-pulse evoked fEPSPs are shown on the top of the graph. Recordings were obtained under Control conditions, 30 min of ISO application and after washing out ISO. **(B)** Aggregate results on PPR are shown for the dorsal and the ventral hippocampus (upper and bottom graph, respectively). **(C)** Time course of the percent change of the PPI showing that application of 10 μM ISO for 30 min does not produce a long-term change in this variable. Representative traces of paired-pulse evoked PSs are shown on the top of the graph. Recordings were obtained under Control conditions, 30 min of ISO application and after washing out ISO. **(D)**. Aggregate results on PPI are shown for the dorsal and ventral hippocampus (upper and lower graph, respectively). Numbers into parenthesis indicate the number of slices/rats used. Asterisks denote statistically significant differences at *p < 0.05, paired *t*-test. Statistically not significant differences are not indicated.

Next, we measured the PPR of synaptic responses (fEPSP2/fEPSP1) evoked by a paired-pulse stimulation to investigate whether ISO affects short-term synaptic plasticity. Figures 2A,B (upper graph) shows that application of ISO in the dorsal hippocampus induced a significant reduction in the ratio fEPSP2/fEPSP1, both acutely (−6.9 ± 1.3%, t_5_ = 7.94, *p* < 0.001) and after washing out the drug [−7.5 ± 2.4%, t_5_ = 4.38, *p* = 0.007; *F*_(2,10)_ = 16.8, *p* < 0.001]. Given the inverse relationship between the transmitter release probability and the magnitude of the ratio fEPSP2/fEPSP1 (Zucker, 1989; Debanne et al., 1996), these results suggested that ISO induced a persistent change in the probability of transmitter release at dorsal hippocampal synapses, even though this change is not reflected as a significant alteration in fEPSP. In contrast, ISO did not significantly affect PPR in the ventral hippocampus either acutely (−4.0 ± 4.9%, t_4_ = 0.7, *p* = 0.523) or after washing out the drug [−2.3 ± 6.1%, t_4_ = 0.176, *p* = 0.869; *F*_(2,8)_ = 0.243, *p* < 0.79] (Figures 2A,B, lower graph).

### 3.5 Effects of β-AR activation on PPI (PS2/PS1)

Previous reports have suggested that E-S potentiation may results from a change in the balance between excitatory and inhibitory inputs onto CA1 pyramidal cells (Abraham et al., 1987; Chavez-Noriega et al., 1989; Lu et al., 2000; Chevaleyre and Castillo, 2003). To assess whether a change in the effectiveness of inhibitory circuitry is involved in E-S potentiation induced by ISO, we measured paired-pulse inhibition (PPI) of the PS by delivering a pair of pulses at short interstimulus intervals (paired-pulse stimulation) to Schaffer collaterals. Under these conditions a reduction in the second PS (PS2) compared with the first PS (PS1) of the pair of responses is produced because of the activation of the local GABAergic inhibition by the first stimulus. We quantified this effect by measuring the PS2/PS1 ratio. We found that ISO application, whether in the dorsal or ventral hippocampus, does not significantly affect the PS2/PS1 ratio either acutely or in the long term (Figures 2C,D). Specifically, we found that the PS2/PS1 ratio did not significantly change in the dorsal hippocampus (32.18 ± 25.7%, t_5_ = 1.69, *p* = 0.151) and the ventral hippocampus (−10.4 ± 14.6%, t_4_ = 1.28, *p* = 0.271) during application of ISO for 30 min. Similarly, we found no significant change in the PS2/PS1 ratio in the dorsal hippocampus [7.47 ± 34.3%, t_5_ = 0.426, *p* = 0.688; *F*_(2,10)_ = 1.7, *p* = 0.232] and the ventral hippocampus [28.32 ± 26.23%, t_4_ = 0.141, *p* = 0.895; *F*_(2,8)_ = 1.63, *p* = 0.255] after washing out ISO. These data support the conclusion that a reduction in the inhibition is unlikely to underlie the E-S potentiation in either of the two segments of the hippocampus.

### 3.6 Effects of prolonged β-AR activation in the dorsal hippocampus

**Figure 3:**
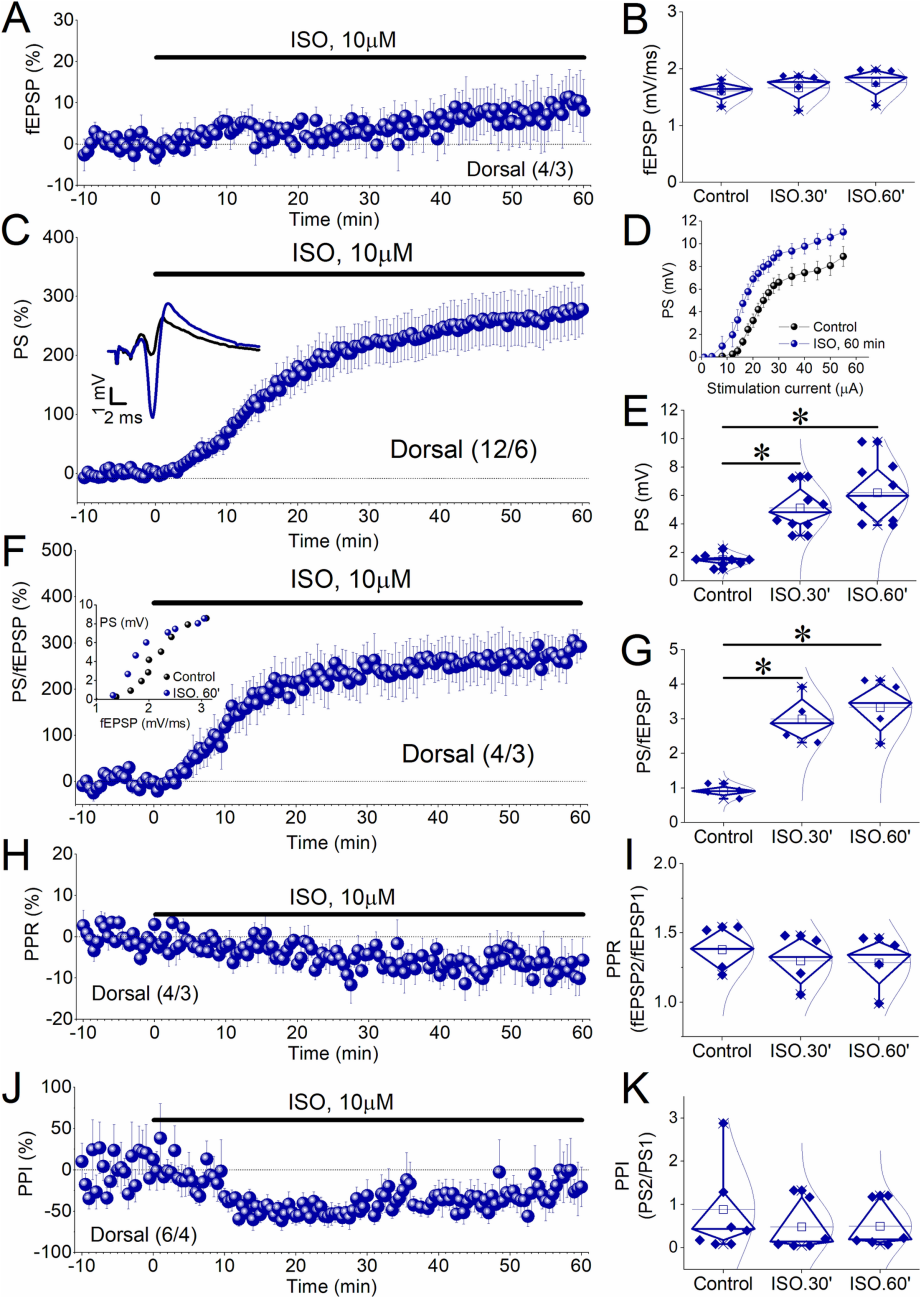
Induction of E-S potentiation by 10 μM ISO applied for 60 min in the dorsal hippocampus. **(A–C)** Time course of percent change of PS showing that application of 10 μM ISO for 60 min induces a non-decremental potentiation of PS in dorsal hippocampal slices like that observed after ISO perfusion for 30 min. Representative traces obtained during Control conditions, and 60 min of ISO application are shown on the top of the graph. **(D)** Aggregate input–output relationship between stimulation current intensity and PS obtained under control conditions (Control), and at the end of ISO perfusion (ISO.60′). **(E)** Box plots of PS obtained before perfusion with ISO (Control), at 30 min of ISO application (ISO.30′), and 60 min of ISO application (ISO.60′) are shown. **(F)** Time course of percent change of PS/fEPSP ratio shows that application of 10 μM ISO for 60 min induces a non-decremental potentiation of PS/fEPSP in dorsal hippocampal slices like that observed after ISO perfusion for 30 min. Example of input–output curve between fEPSP and PS is shown in the insert. **(G)** Box plots of PS/fEPSP ratio obtained from the periods described above. **(H)** Time course of PPR showing the reduction of this variable during the 1 h-long application of ISO in the dorsal hippocampus. **(I)** Box plots of PPR obtained from the three experimental periods (Control, ISO.30′, and ISO.60′). **(J)** Time course of PPI. **(K)** Box plots of PPI obtained from the three experimental periods (Control, ISO.30′, and ISO.60′). Numbers into parenthesis indicate the number of slices/rats used. Asterisks denote statistically significant differences at *p < 0.05 (repeated GLM, pairwise comparisons). Statistically not significant differences are not indicated.

As shown in Figure 1, the increase of PS and PS/fEPSP ratio after washing out ISO was similar to the increase observed during the 30-min application of ISO, in both the dorsal hippocampus (PS, t_8_ = −2.17, *p* = 0.062; PS/fEPSP, t_7_ = −1.45, *p* = 0.19), and the ventral hippocampus (PS, t_6_ = −1.54, *p* = 0.174; PS/fEPSP, t_5_ = −1.95, *p* = 0.109). Data presented in Figures 1C,D indicate that this plateau was reached by the end of ISO application, not before, suggesting that the enhancement of PS could be related to the duration of drug application. Additionally, although the percent change in PS in the dorsal hippocampus does not show a significant difference between 30 and 60 min (pairwise comparisons, *p* = 0.126), there is a slight tendency for it to increase even after the cessation of ISO perfusion (Figure 1C). To explore whether the ISO-induced long-term enhancement of PS and PS/fEPSP in the dorsal hippocampus can be maximized independently of the duration of ISO application, we applied 10 μM ISO for 60 min to a set of dorsal hippocampal slices. The data shown in Figure 3 confirm that the E-S potentiation was independent of the duration of ISO application.

Specifically, application of ISO for 60 min in the dorsal hippocampus did not significantly affect fEPSP [3.2 ± 3.4% and 8.13 ± 6.6% at 30 and 60 min, respectively; *F*_(2,6)_ = 1.68, *p* = 0.263, Figures 3A,B], but it induced a robust long-term potentiation of the PS [263.7 ± 39.7% and 329.0 ± 42.7% at 30 and 60 min, respectively; *F*_(2,14)_ = 44.0, *p* < 0.001, pairwise comparisons, Control-ISO 30 min *p* < 0.001, Control-ISO 60 min *p* < 0.001, ISO 30 min-ISO 60 min *p* = 0.051; Figures 3C–E], and the PS/fEPSP ratio [235.6 ± 44.3% and 269.4 ± 44.1% at 30 and 60 min, respectively; *F*_(2,6)_ = 31.96, *p* < 0.001, pairwise comparisons, Control-ISO 30 min *p* = 0.033, Control-ISO 60 min *p* = 0.028, ISO 30 min-ISO 60 min *p* = 0.376; Figures 3F,G]. Also, the potentiation of the PS and the PS/fEPSP ratio induced by ISO applied for 60 min is comparable with the long-term enhancement observed after applying ISO for 30 min (PS, t_15_ = −0.81, *p* = 0.43; PS/fEPSP, t_10_ = −1.68, *p* = 0.123). We concluded that the induction of long-term potentiation of the PS and the E-S potentiation by ISO is independent of the duration of drug perfusion. In addition, we found that ISO applied for 60 min did not significantly affect the PPR, either at 30 or at 60 min drug application [−6.1 ± 2.1% and − 7.04 ± 4.0% at 30 and 60 min, respectively; *F*_(2,6)_ = 4.21, *p* = 0.072; Figures 3H,I]. However, ISO significantly affected PS2/PS1 ratio (−48.7 ± 10.4% and − 24.3 ± 19.4% at 30 and 60 min, respectively; *χ^2^*(2) = 8.33, *p* = 0.016) by reducing it specifically at 30 min (Z = 1.67, *p* = 0.012) but not at 60 min (Z = 0.833, *p* = 0.447) (Figures 3J, K).

### 3.7 Similar protein expression of β1-AR in the dorsal and the ventral hippocampus

**Figure 4:**
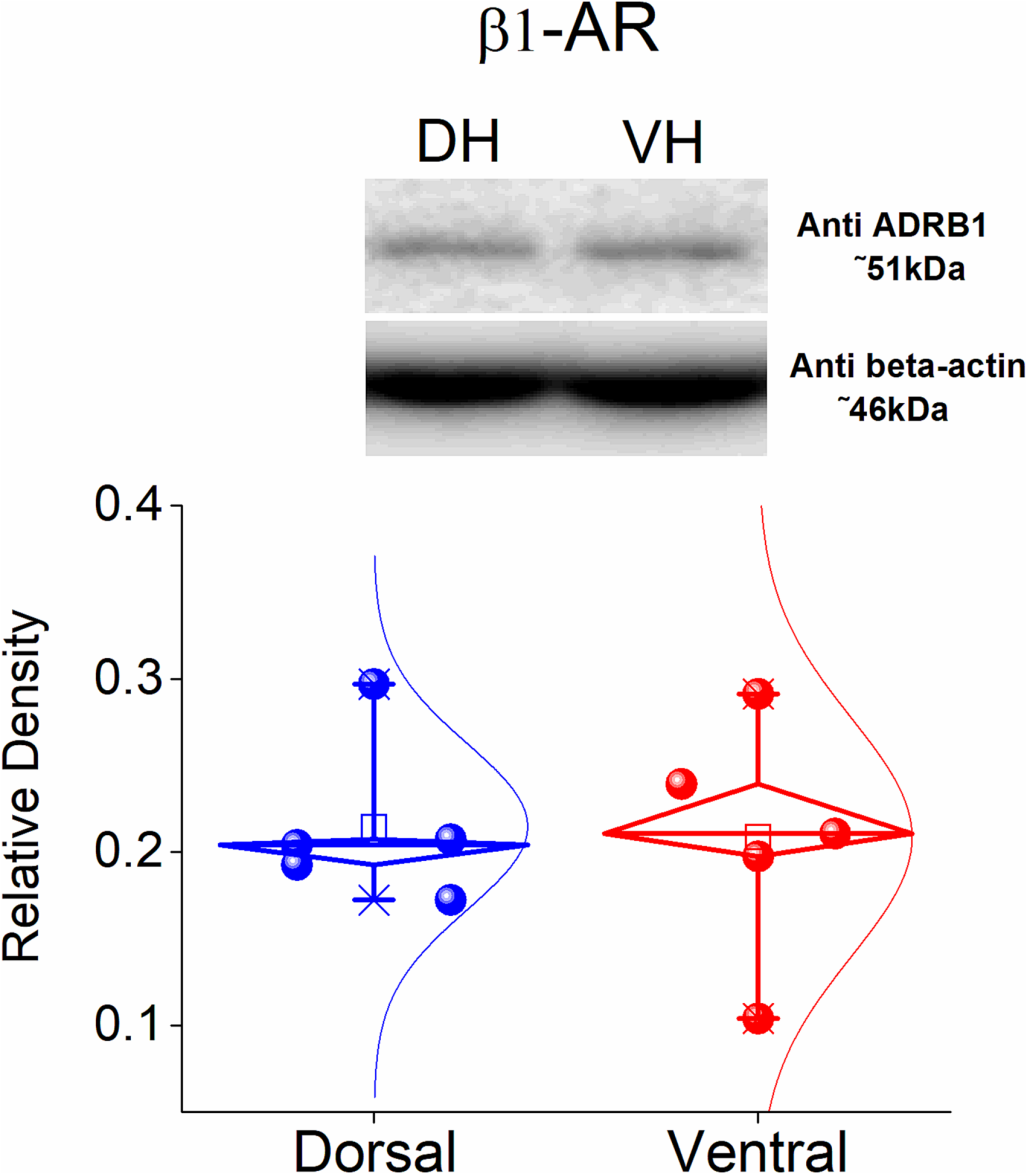
Protein expression of β1-AR in the dorsal and ventral CA1 hippocampal region. Data were obtained from 5 rats. The expression of β1-AR was similar between the two segments of the hippocampus.

Considering the dorsoventral differences in the long-term effects of ISO described above, along with the fact that β1-ARs are more abundantly expressed in the hippocampus compared to β2-ARs (Booze et al., 1993) we investigated whether dorsoventral differences in the expression of β1-ARs might account for the observed differences in plasticity. Thus, we investigated the protein expression of the β1-ARs in the isolated CA1 region of the dorsa land the ventral hippocampus. Using western blotting analysis, we found that β1-AR displays similar expression in the dorsal and the ventral hippocampus (t_8_ = −0.164, *p* = 0.874, *n* = 5, Figure 4). These data showed that the electrophysiological differences found between the dorsal and the ventral hippocampus are not significantly associated with different expression of the β1-AR across the hippocampus.

## 4 Discussion

This study shows that intense activation of β-ARs by isoproterenol induces a long-term enhancement of network excitability, with the magnitude in the dorsal hippocampus being twice that in the ventral hippocampus. This difference cannot be attributed to a difference in expression of β1 adrenergic receptors since they show similar levels in the two parts of the hippocampus.

### 4.1 Possible mechanisms of β-AR effects on E-S potentiation

Previous studies have shown that application of norepinephrine (Dunwiddie and Alford, 1987) or ISO (Fowler and O'Donnell, 1988) can acutely potentiate PS in the CA1 hippocampal region. In addition, some studies showed that ISO induced a potentiation of PS that persisted more than 30 min (Anwyl and Rowan, 1984; Dunwiddie et al., 1992). Also, Mueller et al. (1981) found that norepinephrine produced an increase in PS without a concomitant increase in fEPSP, that lasted at least 10–20 min following washout of norepinephrine. Application of norepinephrine in the dentate gyrus *in vivo* (Neuman and Harley, 1983) or *in vitro* (Lacaille and Harley, 1985; Heginbotham and Dunwiddie, 1991) produced a persistent increase in PS accompanied by a lower potentiation or no change of fEPSP, that lasted more than 30 min. In a later study, potentiation of PS without concomitant change in fEPSP, has been observed in CA1 region following co-application of ISO with an agonist of metabotropic glutamate receptors (Gereau and Conn, 1994). However, in several of these studies data on fEPSP were scarce and/or evoked responses were monitored for a rather limited time of drug washout. The present is the first study to clearly show that β-AR activation produces a long-lasting E-S potentiation with greater magnitude in the dorsal compared with the ventral hippocampus.

Several mechanisms have been proposed to underlie or contribute to E-S potentiation, with the main emphasis having been given on mechanisms of intrinsic excitability and synaptic inhibition. Notably, previous reports have been suggested that E-S potentiation may result from an increase in intrinsic excitability (Hess and Gustafsson, 1990; Asztely and Gustafsson, 1994; Jester et al., 1995; Fink and O'Dell, 2009), or a decrease in feed-forward inhibition (Abraham et al., 1987; Chavez-Noriega et al., 1989; Lu et al., 2000; Chevaleyre and Castillo, 2003). We found that E-S potentiation induced by ISO was not accompanied by any significant change in PPI, suggesting that long-lasting reduction in inhibition is unlikely to underlie E-S potentiation induced by β-AR activation, as has also been suggested for E-S potentiation induced by a mild 5 Hz stimulation pattern (Fink and O'Dell, 2009).

The role that β-ARs play in modulating synaptic LTP induced by high-frequency stimulation or patterned stimulation at the theta frequency is well established (Stanton and Sarvey, 1984; Thomas et al., 1996; Katsuki et al., 1997; Brown et al., 2000; Straube et al., 2003; Gelinas and Nguyen, 2005; Gelinas et al., 2008; Qian et al., 2012; Jedrzejewska-Szmek et al., 2017; Papaleonidopoulos and Papatheodoropoulos, 2018; Jami et al., 2023), and crucial signaling steps in this action of β-ARs include Gs protein-dependent activation of adenyl cyclase, elevation of intracellular cAMP, activation of PKA and inhibitor-1, that inhibits protein phosphatase activity (O'Dell et al., 2015). Nevertheless, the specific role of β-ARs and the downstream signaling pathways recruited to mediate their effect on synaptic plasticity appear to differ depending on the specific context of neuronal activation. For instance, although activation of β-ARs in the hippocampus facilitates the induction of synaptic LTP by high-frequency stimulation, β-ARs are not required for this form of LTP (Murchison et al., 2004). In contrast, β-ARs are required for the induction of synaptic LTP by stimulation patterns that alone have minor effects on synaptic strength (Thomas et al., 1996; Katsuki et al., 1997; Brown et al., 2000; Gelinas and Nguyen, 2005; Qian et al., 2012; Papaleonidopoulos and Papatheodoropoulos, 2018; Jami et al., 2023). Moreover, the mechanisms of E-S potentiation may differ from those involved in the induction of synaptic LTP, as E-S potentiation reflects an increased coupling between the dendritic synapses and the somatic region - the axon initial segment - where the action potential originates. Therefore, it can be expected that the mechanisms of E-S potentiation are dendritically localized at points downstream of the synaptic region (Fink and O'Dell, 2009), leading to a sustained increase in excitability. These mechanisms may ultimately be related to changes in the properties, number, or distribution of ion channels expressed on the neural cell membrane (Zhang and Linden, 2003). Interestingly, mild stimulation patterns based on the frequency of theta rhythm, namely a sequence of 15 pulses at 5 Hz delivered at the Schaffer collaterals, induce a pure form of E-S potentiation at CA1 synapses that require the activity of protein phosphatases, including calcineurin, but is independent of protein kinase activity (Fink and O'Dell, 2009).

Potassium channels in the dendrites, including voltage-gated A-type potassium channels (that belong to Kv channel family), and small conductance, Ca^2+^-activated SK2 potassium channels, strongly limit membrane depolarization and neuronal excitability (Magee et al., 1998; Bloodgood and Sabatini, 2007; Kim and Hoffman, 2008; Liu et al., 2017), and the activity of protein phosphatases plays an important role in modulating these potassium channels. For instance, protein phosphatase activity downregulates Kv2.1 channels (Peretz et al., 2000), and the phosphatase calcineurin promotes the internalization of these potassium channels leading to hyperexcitability (Misonou et al., 2004). Furthermore, recent evidence shows that calcineurin inhibits Kir4.1/Kir5.1 potassium channels (Zhang et al., 2023).

Interestingly, β-AR activation downregulates A-type (Kv4.2) and SK potassium channels (Hoffman and Johnston, 1998; Johnston et al., 1999; Yuan et al., 2002; Faber et al., 2008; Maingret et al., 2008), facilitating the LTP induction by high-frequency stimulation. For instance, β2 adrenergic receptors inhibit Kv1.1 channels, thereby increasing CA1 pyramidal cell excitability (Liu et al., 2017). Accordingly, persistent downregulation of potassium ion channels - activation of which tends to repolarize or hyperpolarize the cell membrane - through changes in their properties, number, or distribution, could lead to a sustained potentiation of neuronal excitability. Interestingly, the expression of a subtype of Kv potassium channels (Kv4.2) has been found increased in the dorsal than the ventral hippocampus (Marcelin et al., 2012), suggesting that an increased number of potassium channels that could be downregulated by the action of β-ARs may be involved to the dorsoventral difference in E-S potentiation. Future research should examine the involvement of protein phosphatase activity in the induction of E-S potentiation and clarify the specific roles of the subtypes of β-ARs in this process. Nevertheless, the current evidence suggests a non-differentiated contribution of β1 receptors in E-S potentiation, as their levels appear similar in both sections.

### 4.2 Functional implications for the dorsal and the ventral hippocampus

The present evidence shows that both dorsal and ventral hippocampus are well capable for β-AR induced E-S potentiation, however, their response to β-AR activation differs quantitatively. It has been previously shown that β-ARs are involved in contextual fear memory (Murchison et al., 2004; Izquierdo et al., 2016). Both, dorsal and ventral hippocampus are involved in contextual fear memory, however, they play different roles (Maren et al., 2013; Xu et al., 2016). Accordingly, the present evidence may suggest that E-S potentiation induced by relatively intense β-AR activation may be implicated in the processing of events occurring under conditions of increased emotional arousal, playing, however, different roles in the two segments of the hippocampus. E-S potentiation in the dorsal hippocampus may contribute to processing the spatial aspects of contextual information (Maren et al., 2013), while E-S potentiation in the ventral hippocampus may be involved in contextual fear generalization (Xu et al., 2016).

Considering that neuronal intrinsic excitability may significantly contribute to E-S potentiation (Hess and Gustafsson, 1990; Asztely and Gustafsson, 1994; Jester et al., 1995; Fink and O'Dell, 2009), E-S potentiation may serve various functions, including modulation of neuronal input–output function (Debanne et al., 2019), a fundamental computational feature of neural function (Koch, 2004), as well as temporal processing and homeostasis (Zhang and Linden, 2003; Debanne et al., 2019). Furthermore, it has been speculated that lasting intrinsic plasticity might be involved in forming a hyperexcitable state that serves as a neural substrate for the consolidation or adaptive generalization of memories (Zhang and Linden, 2003). This may be related to the limited E-S potentiation shown by the ventral hippocampus. The relative difficulty of the ventral hippocampus for E-S potentiation appears to be related to its generally limited capacity for long-term synaptic plasticity, which in turn may be linked to the organization and functions of this hippocampal region. Synaptic plasticity changes the input–output relationship in neuronal networks and tends to destabilize their activity (Turrigiano, 1999). E-S potentiation, being a condition of net enhancement of the neuronal output, is expected to have an even greater impact on the input–output relation, tending to destabilize the activity of the neuronal network in a strong way.

The ventral hippocampus is constitutively characterized by a relatively increased neuronal excitability that may serve its specific functional roles since this segment of the hippocampus functions effectively under normal conditions. However, under conditions that increase the level of excitation or directly enhance network excitability, such as E-S potentiation, the ventral hippocampus may transition into a state of hyperexcitability, potentially disrupting its physiological function. This disruption is particularly evident during epileptic seizures, to which the ventral hippocampus is especially vulnerable; see (Papatheodoropoulos, 2018). The lower ability of the ventral hippocampus for E-S potentiation may therefore reflect a requirement for keeping excitability into a relatively limited range, thereby preventing the transition of the local network toward hyperexcitability, while retaining the ability to process and store information through this mechanism. Such a role for E-S potentiation in the ventral hippocampus is compatible with the postulated role that intrinsic plasticity may play to homeostasis in neuronal networks (Turrigiano, 1999; Zhang and Linden, 2003; Debanne et al., 2019).

It is also interesting to note that under cholinergic activation, norepinephrine suppresses hippocampal excitability (Valero-Aracama et al., 2021), which contrasts with the ISO-induced long-term enhancement of excitability observed here. Both actions seem to involve intrinsic cellular mechanisms, suggesting that norepinephrine’s effects may be dynamically influenced by the neurochemical context. Consequently, the relatively limited ability of β-ARs to produce E-S potentiation in the ventral hippocampus may relate to its specific neurochemical environment; for example, genes associated with cholinergic transmission are highly expressed in the ventral hippocampus (Lee et al., 2017). Future studies could explore how cholinergic transmission interacts with β-AR activity to affect E-S potentiation in the dorsal and ventral hippocampus and examine the involvement of various receptor subtypes in these processes.

Interestingly, while β-ARs are crucial in the ventral hippocampus for inducing synaptic LTP under otherwise subthreshold stimulation conditions (Papaleonidopoulos and Papatheodoropoulos, 2018; Jami et al., 2023), the present findings indicate that strong β-AR activation alone can produce a sustained increase in local network excitability, with a more pronounced effect in the dorsal than in the ventral hippocampus. This suggests that β-ARs may adjust their roles in both synaptic and intrinsic plasticity along the dorsoventral axis of the hippocampus, potentially modulating their impact based on the contextual and emotional significance of the experience being encoded.

Thus, it is plausible to suggest that the dorsal hippocampus may be more deeply engaged during highly stressful or dangerous situations that require enhanced alertness and hyper-vigilance, facilitating a “fight-or-flight” response. In such states, individuals must rapidly process and retain spatial information to navigate their environment and respond effectively to immediate threats (Sara, 2009; Mather and Sutherland, 2011). In contrast, the ventral hippocampus, with its greater sensitivity to adrenergic modulation of long-term plasticity under moderately increased glutamatergic input (Papaleonidopoulos and Papatheodoropoulos, 2018; Jami et al., 2023), may play a more prominent role in encoding emotionally salient information, particularly in the context of novelty detection (Bouret and Sara, 2005).

## Data Availability

The raw data supporting the conclusions of this article will be made available by the authors, without undue reservation.
